# Influence of TMAO as co‐solvent on the gelation of silica‐PNIPAm core‐shell nanogels at intermediate volume fractions

**DOI:** 10.1002/cphc.202000114

**Published:** 2020-06-02

**Authors:** Lara Frenzel, Irina Lokteva, Michael Koof, Suresh Narayanan, Gerhard Grübel, Felix Lehmkühler

**Affiliations:** ^1^ Deutsches Elektronen-Synchrotron DESY Notkestr. 85 22607 Hamburg Germany; ^2^ The Hamburg Centre for Ultrafast Imaging Luruper Chaussee 149 22761 Hamburg Germany; ^3^ Advanced Photon Source Argonne National Laboratory Argonne Illinois 60439 United States

**Keywords:** Colloids, Gels, PNIPAm, Small-angle X-ray scattering, X-ray photon correlation spectroscopy

## Abstract

We study the structure and dynamics of poly(N‐isopropylacrylamide) (PNIPAm) core‐shell nanogels dispersed in aqueous trimethylamine N‐oxide (TMAO) solutions by means of small‐angle X‐ray scattering and X‐ray photon correlation spectroscopy (XPCS). Upon increasing the temperature above the lower critical solution temperature of PNIPAm at 33 °C, a colloidal gel is formed as identified by an increase of *I*(*q*) at small *q* as well as a slowing down of sample dynamics by various orders of magnitude. With increasing TMAO concentration the gelation transition shifts linearly to lower temperatures. Above a TMAO concentration of approximately 0.40 mol/L corresponding to a 1 : 1 ratio of TMAO and NIPAm groups, collapsed PNIPAm states are found for all temperatures without any gelation transition. This suggests that reduction of PNIPAm‐water hydrogen bonds due to the presence of TMAO results in a stabilisation of the collapsed PNIPAm state and suppresses gelation of the nanogel.

## Graphical abstract

**The gelation** of silica‐PNIPAm core‐shell nanogels around 40 °C at intermediate volume fractions is found to be dependent on the concentration of TMAO as co‐solvent. Above a 1 : 1 molar ratio of NIPAm units and TMAO the particle are in a collapsed state and gelation is suppressed.

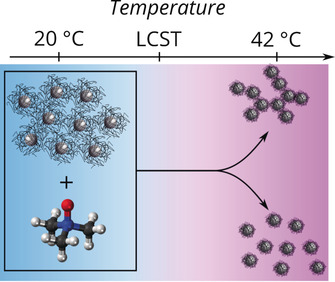

## 1 Introduction

Stimuli‐responsive polymer micro‐ and nanogels are cross‐linked polymeric particles that are swollen with solvent molecules. Upon a particular stimulus, e. g., pressure or temperature changes, the particles undergo a volume phase transition changing their configuration by releasing solvent molecules.^1^ Within this particle class, microgels consisting of poly(N‐isopropylacrylamide) (PNIPAm) are widely studied, as seen in a variety of recent reviews.^2^, ^3^, ^4^, ^5^ PNIPAm particles dispersed in water show a reversible volume phase transition at a lower critical solution temperature (LCST) of 33 °C. Below this LCST, the particles are swollen with water. They become hydrophobic above this temperature, releasing the water and forming a collapsed state.

This process has been widely studied, predominately by light scattering determining the particle deswelling as a function of temperature.^6^, ^7^, ^8^ Although the majority of these studies focuses on single particle properties, there has been a growing interest on higher particle packings. In particular, the use of PNIPAm micro‐ and nanogel particles for colloidal studies where the volume fraction is varied in‐situ by changing the temperature and thus the particle size, has been questioned because the interaction potential changes as well.^9^ Furthermore, interpenetration of PNIPAm particles may become possible which allows reaching very high volume fractions, even above 100 vol.%.^10^, ^11^, ^12^ While below the LCST PNIPAm micro‐ and nanogels behave like a soft colloidal fluid, the increase of attractive interactions in the collapsed state above the LCST results in the formation of a colloidal gel.^13^, ^14^, ^15^, ^16^, ^17^, ^18^ Recently, we studied the structure and dynamics of densely‐packed core‐shell nanoparticles consisting of a silica core and a PNIPAm shell by means of coherent X‐ray scattering.^19^, ^20^ At low volume fractions no gelation was observed, while gelation shows a temperature dependence at intermediate concentrations. Nevertheless, the gelation temperature was found to be around *T*_gel_≈37 °C for high volume fraction, well above the LCST for these states.^20^

The proximity of the LCST to biological‐relevant temperatures makes PNIPAm a promising material for applications in medicine and technology.^1^, ^2^, ^5^ Therefore, the impact of co‐solvents on PNIPAm properties, e. g., tuning the LCST becomes important. As PNIPAm can be dissolved as well in many alcohols, many experimental and theoretical investigations focus on water‐methanol mixtures. They report a co‐nonsolvency,^21^, ^22^, ^23^, ^24^, ^25^, ^26^ i. e., a reduction of the LCST below values found for the pure solvents. A reduction of the LCST has been reported for many other co‐solvents, such as urea,^27^, ^28^ acetone,^29^ and ethanol.^30^ The reason for the reductions is unknown. For alcohol, a localization of alcohol molecules close to the polymer interface has been reported. Further, the reduction of the LCST is suggested to be a consequence of the release of water molecules from the PNIPAm hydrophobic moiety favoring the aggregation of ethanol molecules in the vicinity of the polymer.^30^ Another co‐solvent that was found to have an impact on the swelling of PNIPAm is the osmolyte trimethylamine N‐oxide (TMAO). TMAO is known to stabilize proteins,^31^, ^32^ e. g., against high pressure.^33^, ^34^ Despite many studies in the last decades, the exact stabilization mechanism is still unclear, potentially TMAO stabilizes water hydrogen bonds^35^, ^36^ and hence the protein indirectly. Likewise, the addition of TMAO as co‐solvent to aqueous PNIPAm micro‐ and nanogels leads to a stabilisation of globular PNIPAm states, and consequently to an effective reduction of the LCST in the presence of TMAO.^37^, ^38^, ^39^, ^40^ These observations have been rationalized by a formation of hydrogen bonds between TMAO and water molecules that are bound to PNIPAm.^37^ Other studies concluded an increase in the magnitude of solvent‐excluded volume effects because PNIPAm interacts preferentially with water.^40^ Furthermore, studies on polystyrene reported a increased binding affinity of TMAO with collapsed conformation of polystyrene compared to the extended one,^41^ which has been found as well for PNIPAm.^38^ However, these investigations focused so far on low PNIPAm concentrations, i. e., the impact of co‐solvents on the volume phase transition of PNIPAm micro‐ and nanogels in the single‐particle limit. It is unclear how TMAO affects high volume fractions, in particular where gelation has been found.

Here, we report results on the structure and dynamics of silica‐PNIPAm core‐shell nanogels at a volume fraction of $\phi_{\mathrm{eff},20}=0.14$ at 20 °C dispersed in aqueous TMAO solutions. We employ coherent X‐ray scattering in small‐angle scattering geometry to cover the relevant length scales of the colloidal system. The dynamics are extracted by means of X‐ray Photon Correlation Spectroscopy (XPCS). Upon increasing the temperature above the LCST, we find the gelation transition which has been reported recently for this system.^19^, ^20^ With increasing TMAO concentration the gelation transition shifts to lower temperatures. For the highest TMAO concentration studied in this work of 0.52 mol/L the system does not show any indication of gelation and rather behaves diffusive over the studied temperature range. This suggests that a 1 : 1 ratio of TMAO and NIPAm groups reduces the number of PNIPAm‐water hydrogen bonds and results in a stabilisation of the collapsed PNIPAm state and suppression of gelation at intermediate volume fractions.

## 2 Results and Discussion

Illuminating a disordered soft‐matter sample with a (partially) coherent X‐ray beam generates a grainy diffraction pattern, the so‐called speckle pattern. Analogous to dynamic light scattering (DLS) the dynamics of a sample can be studied by tracking the time evolution of the speckles via the intensity‐intensity correlation function^42^, ^43^, ^44^, ^45^

(1) $$g_{2}\left(q,\tau \right)=\frac{\left\langle I\left(q,t\right)I\left(q,t+\tau \right)\right\rangle }{\left\langle I\left(q,t\right)\right\rangle },$$

probed at the modulus of the wave vector transfer $q=\left|q\right|=\frac{4\pi }{\lambda }\sin\left(\theta /2\right)$ with wave length *λ* and scattering angle *θ*. The average in Eq. 1 is performed over the experimental time *t* and detector pixels with the same *q*. This correlation function is connected to the intermediate scattering function that includes all information of the time evolution of the sample via

(2) $$g_{2}\left(q,\tau \right)=1+\beta {\left|f\left(q,\tau \right)\right|}^{2}.$$

The speckle contrast *β* is defined by experimental parameters, such as the degree of coherence, the beam size, and the detector pixel size. For soft matter systems as studied here, the correlation function can be described by a Kohlrausch‐Williams‐Watts (KWW) function^42^, ^43^, ^44^, ^45^

(3) $$g_{2}\left(q,\tau \right)=1+\beta \;\exp(-2\left({\Gamma \tau )}^{\gamma }\right).$$

Here, the shape of the *g*_2_‐function is defined by the KWW exponent *γ*. The relaxation rate $\Gamma \left(q\right)$ relates to the relaxation time *τ_c_* via $\Gamma \left(q\right)=1/\tau_{c}\left(q\right)$ and typically follows a power law as function of *q* as $\Gamma \left(q\right)\propto q^{p}$ . The type of dynamics can be qualified by both exponents, e. g., diffusion is characterized by γ=1 and $\Gamma =Dq^{2}$ , with the Stokes‐Einstein diffusion constant $D=\frac{k_{B}T}{6\pi \eta R}$ , Boltzmann's constant *k*_B_, temperature *T*, solvent viscosity *η* and particle radius *R*.

In order to investigate the influence of TMAO on the temperature‐dependence of the silica‐PNIPAm nanogel, we compare the average sample structure obtained by SAXS with the dynamics revealed simultaneously by XPCS.

### 2.1 SAXS results

**Figure 1:**
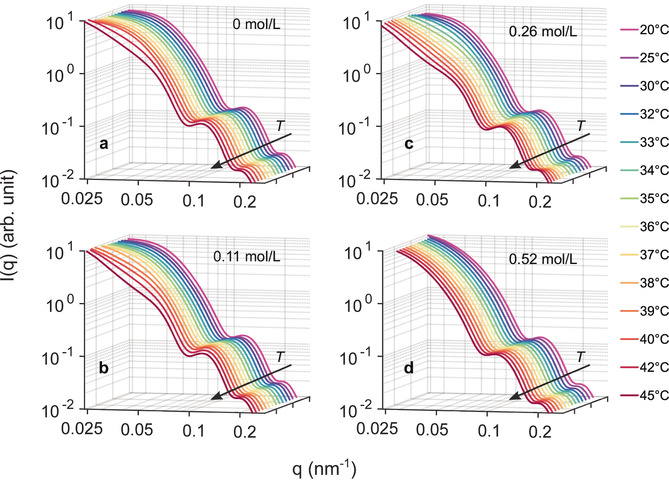
Temperature dependence of *I(q)* for different TMAO concentrations: (a) pure water‐PNIPAm dispersion, (b) 0.11 mol/L TMAO, (c) 0.26 mol/L TMAO, and (d) 0.52 mol/L TMAO.

The SAXS intensity *I*(*q*) is shown in Figure 1 for four TMAO concentrations and temperatures between 20 °C and 45 °C. The 2D speckle patterns were averaged for each state before obtaining *I*(*q*) via azimuthal integration. For 0 mol/L TMAO, the data follow our previous results.^19^, ^20^ At low temperature the *I*(*q*) resembles the form factor of spherical core‐shell particles, i. e., the intensity drops with increasing *q* and shows oscillations. With increasing temperature, the slope at low *q* changes resulting in a steeper increase for q→0 . This behaviour is typically connected to the formation of large‐scale structure, such as agglomerates, within the sample and was rationalized by a change from repulsive to attractive interaction in PNIPAm systems above the LCST.^13^, ^19^, ^20^, ^46^, ^47^ For TMAO concentrations up to *c*=0.26 mol/L similar results are obtained. However, the upturn of *I*(*q*) for q→0 sets in at lower temperatures and appears slightly stronger at 45 °C. In contrast, for *c*=0.52 mol/L TMAO the *I*(*q*) do not change significantly with *T*. Compared to the lower concentrations, the *I*(*q*) drop at low *q* is furthermore steeper at low temperatures. This indicates a different structure at this concentration that seems to be independent of temperature.

**Figure 2:**
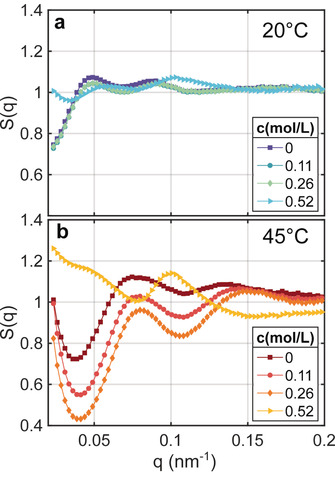
Structure factors *S*(*q*) of the dispersions shown in Figure 1 at (a) 20 °C and (b) 45 °C.

To gain more details on structural changes as a function of TMAO concentration and temperature, we calculated effective structure factors *S*(*q*) by normalization of *I*(*q*) by the form factor *P*(*q*) which was measured from diluted colloidal dispersions. The structure factors are shown in Figure 2 for 20 °C and 45 °C. These temperatures represent both the swollen and collapsed state of the PNIPAm shell. At 20 °C (Figure 2a), structure factors with a maximum at *q*_max_≈0.048 nm^−1^ can be found for c≤0.26 mol/L. As they vary only slightly with *c*, we conclude that TMAO has only very weak impact on the structure of the dispersion. For *c*=0.52 mol/L *S*(*q*) shows only weak modulations around 1. This suggests that the structure of the dispersion resembles basically the structure of the form factor sample with $S\left(q\right)\approx 1$ which is representative for dilute colloidal dispersions. Furthermore, the slight upturn for q→0 nm^−1^ and the weak modulations may suggest appearance of a small number of agglomerates due to attractive polymer‐polymer interactions.^7^, ^47^, ^48^

At elevated temperatures where *I*(*q*) shows the upturn at low *q*, we found a distinct *c*‐dependence of *S*(*q*) (Figure 2b). For q→0 the *S*(*q*) indeed grows, in particular for the low TMAO concentrations. The peak position shifts to $q_{\max}\approx 0.077$ nm^−1^ which indicates a reduction of the average next‐neighbour distance as well as the formation of agglomerates as mentioned above. Furthermore, the *S*(*q*) shows different amplitudes for q<0.15 nm^−1^, i. e., it decreases with increasing *c*. The structure factor at c=0.52 mol/L shows a more complex shape with a peak at $q_{\max}\approx 0.1$ nm^−1^ and modulations for q→0 . Compared to the lower TMAO concentrations this suggests a closer packing with different types of agglomerates, however, the effects are too weak to draw a final conclusion.

Altogether the evolution of *S*(*q*) with *T* indicates a transition from repulsive interactions – represented by the *S*(*q*) for c≤0.26 mol/L in Figure 2a – towards attractive interactions at high temperatures. This is in agreement with our recent studies on pure silica‐PNIPAm dispersions.^19^, ^20^ In contrast, at high TMAO concentrations the interaction potential seems to be less affected by the temperature change, assuming an unchanged sample structure. Nevertheless, we would like to note that a more detailed analysis of *S*(*q*) will need an extended *q*‐range towards lower *q*, e. g., using ultra‐small‐angle X‐ray scattering geometries^49^, ^50^ to capture the formation and type of large‐scale clusters in the sample.

### 2.2 XPCS results

**Figure 3:**
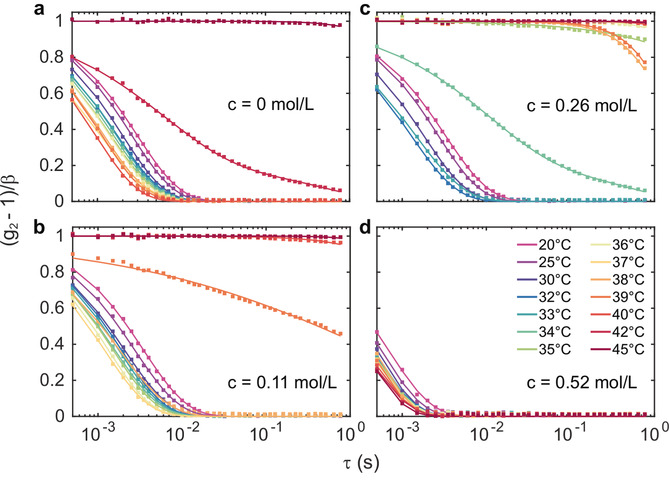
Intensity correlation functions *g*_2_ for different TMAO concentrations: (a) pure water‐PNIPAm dispersion, (b) 0.11 mmol/L TMAO, (c) 0.26 mol/L TMAO, and (d) 0.52 mol/L TMAO. The symbols represent the measured *g*_2_ functions, solid lines are fits following Eq. 3.

The dynamics of the core‐shell particles have been studied by means of XPCS. Correlations from series of speckle patterns have been analyzed following Eq. 1. Due to the short exposure and thus the limited count rate in the speckle patterns, the analysis was limited to typically q<0.046 nm^−1^. The resulting *g*_2_‐functions are shown in Figure 3 for different TMAO concentrations. The *g*_2_ functions of the pure silica‐PNIPAm sample suggest first a speeding‐up upon heating up to *T*=40 °C. This is connected to the reduction of solvent viscosity *η* at higher temperatures and consequently a reduction of the relaxation time *τ_c_*. Upon further heating to *T*_gel_=42 °C, the sample slows down and becomes almost static over the studied time range at 45 °C. In general, the correlation functions are modeled by a single, stretched exponential decay following Eq. 3. At *T*_gel_=42 °C, two relaxation processes are used to model the data^51^ and to capture the appearance of the slow relaxation process. This behaviour was reported recently for different pure silica‐PNIPAm systems and has been related to the formation of a colloidal gel upon heating.^19^, ^20^ Most interestingly, the gelation transition at *T*_gel_ takes place well above the LCST – in this case at approximately 10 °C higher temperatures. This matches the phase behaviour found for similar volume fractions.^20^

For low TMAO concentrations (c≤0.26 M), the *g*_2_‐functions show a similar behaviour. However, the slow‐down of dynamics appears to happen at lower gelation temperatures *T*_gel_ compared to the pure sample, i. e., at approximately 38.5 °C for 0.11 mol/L TMAO and 34 °C for 0.26 mol/L TMAO. In contrast, the sample at c=0.52 mol/L TMAO only shows the speed‐up with increasing temperature without indication of gelation. This is in line with the observation of the *I*(*q*), where as well no structural transition was found.

**Figure 4:**
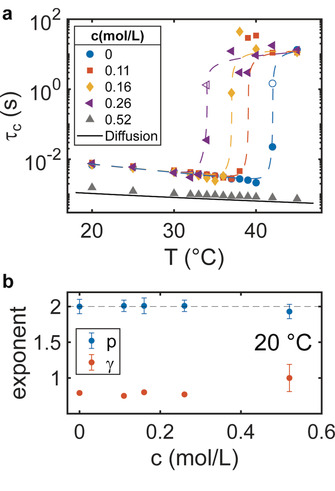
(a) Relaxation times *τ_c_* as a function of temperature for all TMAO concentrations *c* obtained by XPCS. The dashed lines are guides to the eyes, the open symbols are used for the slow process when two relaxation processes are present. The solid black line is the calculated diffusion time for a spherical particle with 75 nm radius in a liquid with viscosity of a 0.52 mol/L TMAO solution. (b) Exponents *p* and *γ* as a function of TMAO concentration at *T*=20 °C.

In the next step we take a closer look on the relaxation times and the type of dynamics. In Figure 4a the relaxation times extracted from the KWW fits are shown for q=0.023 nm^−1^ for all studied TMAO concentrations. Here, results of the intermediate concentration of c=0.16 mol/L are added as well. For $T\leq 32^{\circ }$ C and c≤0.26 mol/L, the relaxation times match each other well. Increasing the temperature further, the relaxation times increase by several orders of magnitude. Note that the statistical accuracy is limited for $\tau_{c}\geq 5$ s due to the limited exposure time on the samples. Nevertheless, few values could be modelled from longer XPCS series measured at some temperatures. The onset of slow‐down at *T*_gel_ is clarified by the dashed lines. In contrast, *τ_c_* for c=0.52 mol/L TMAO shows a continuous decrease with *T*. This follows in general the expectations for diffusion of nanoparticles in a liquid. Therefore, we calculated the diffusive relaxation time for particles of R=75 nm, i. e., the collapsed state, shown as solid line in Figure 4a. The viscosity of water‐TMAO as solvent was extrapolated from literature values^52^ and was corrected for the volume fraction of hard spheres.^53^ We relate the difference between *τ_c_* for the sample at 0.52 mol/L TMAO and the calculation for diffusion of a factor of approximately 1.3 deviating from the hard sphere case to an effective slow‐down of dynamics because of non‐zero particle‐particle interaction. This suggests that the sample is in a collapsed state for all temperatures. Furthermore, this is in line with the *S*(*q*) data at low temperatures (see Figure 2a), where indications were found for few agglomerates due to polymer‐polymer interactions typically reported for collapsed PNIPAm states.^13^, ^46^, ^47^

The type of dynamics is further studied by the exponents *p* describing the *q*‐dependency of *τ_c_* and *γ* which is a measure of the shape of the *g*_2_‐function. Both exponents are given in Figure 4b for *T*=20 °C. Due to the limited statistical accuracy above *T*_gel_, the exponents cannot be extracted. For all concentrations we found p=2 and γ≈0.8 , which is close to the expectations for diffusion of p=2 and γ=1 . The slightly lower values of *γ* suggest sub‐diffusive or heterogeneous dynamics^54^, ^55^, ^56^ and have been reported before for PNIPAm systems.^19^

**Figure 5:**
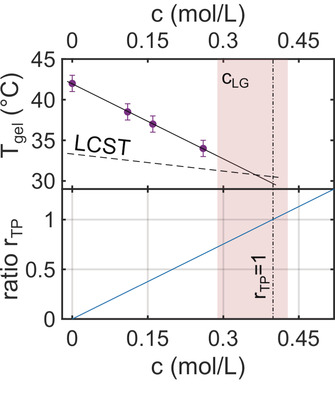
Top: Gelation temperature *T*_gel_ in dependence of TMAO concentration. The dashed line marks the *c*‐dependence of the LCST,^37^, ^39^ the solid line is a linear fit to the data. The light red area represents the region where both lines cross at $c_{\mathrm{LG}}=\left(0.36\pm 0.07\right)$ mol/L. Bottom: TMAO‐NIPAm unit molar ratio *r*_TP_ as a function of *c*. The dashed‐dotted line represents $r_{\mathrm{TP}}=1$ at $c_{\mathrm{TP}}=0.40$ mol/L.

The shift of *T*_gel_ observed in Figure 4a is highlighted in Figure 5 (top). The error bar for *T*_gel_ was set to ΔT =1 °C which equals to the temperature steps used. Remarkably, we found a linear relation between *T*_gel_ and *c* with a rate of $\frac{dT_{\mathrm{gel}}}{dc}=-30.8^{\circ }$ CL/mol. Compared to *T*_gel_, the reduction of the LCST with TMAO concentration is less pronounced (dashed line in Figure 5 (top)). Here, a linear model based on data for TMAO concentrations up to 1 mol/L reported in literature was used,^37^, ^39^ leading to a rate of $-7.0^{\circ }$ CL/mol. Both lines intercept at $c_{\mathrm{LG}}=\left(0.36\pm 0.07\right)$ mol/L which may suggest that no gelation takes place above this value. For $c>c_{\mathrm{LG}}$ the volume phase transition as prerequisite for gelation will not have taken place. In fact, our measurement at c=0.52 mol/L does not show any indication for gelation.

These observation are now discussed in the framework of the TMAO‐PNIPAm interaction. Therefore, we calculated the molar ratio *r*_TP_ of TMAO molecules and NIPAm groups of the PNIPAm shells. The results are shown in Figure 5 (bottom). We found $r_{\mathrm{TP}}=1$ , i. e., equal amount of TMAO molecules and NIPAm groups, for $c_{\mathrm{TP}}=\left(0.40\pm 0.05\right)$ mol/L. Interestingly, this coincides with *c*_LG_, for larger TMAO concentrations PNIPAm is collapsed and consequently no gelation can be observed, as observed for c=0.52 mol/L. We interprete this observation by the TMAO‐PNIPAm interaction. TMAO was reported to reduce the number of hydrogen bonds between PNIPAm and water^37^ and in addition preferentially bind to the collapsed PNIPAm state.^38^, ^41^ Our results suggest that a 1 : 1 molar ratio of TMAO and NIPAm groups fosters the collapse of PNIPAm and supresses gelation at intermediate volume fractions.

## 3 Conclusions

In summary, we measured coherent SAXS patterns of silica‐PNIPAm core‐shell nanogels with the presence of TMAO as co‐solvent. At low TMAO concentrations, *I*(*q*) shows an upturn for q→0 at temperatures above the LCST. This suggests a transition from repulsive to attractive interactions as reported previously as a fingerprint for gel formation in this system.^19^ The sample at the highest TMAO concentration studied (0.52 mol/L) shows the *I*(*q*) of a dilute colloidal fluid with weak *S*(*q*) contribution only. This is supported by the XPCS results, where the dynamics of this sample at 0.52 mol/L are diffusive over the whole temperature range. The combination of SAXS and XPCS results suggests, that the PNIPAm shell is collapsed for all temperatures, in particular enforcing a volume phase transition by the presence of TMAO. In contrast, the relaxation times obtained for c≤0.26 mol/L increase by several orders of magnitude at certain temperatures above the LCST. This is in agreement with the *I*(*q*) and our previous studies on pure silica‐PNIPAm core‐shell systems where this upturn was a consequence of a gelation transition. Here, the gelation temperature *T*_gel_ depends on the TMAO concentration *c*. Above a particular TMAO concentration, the particles stay in the collapsed state and do not form a gel state.

These observations can be rationalized as follows: First, the gelation of the nanogel takes place at approximately 42 °C which is in agreement with our previous observation.^20^ Second, the gelation temperature is found to be reduced linearly with increasing TMAO concentration up to 0.26 mol/L, much stronger than the decrease of the LCST. Third, above a threshold TMAO concentration that corresponds to a 1 : 1 ratio of TMAO and NIPAm groups, we found collapsed PNIPAm states for all temperatures and no gelation transition. This indicates that one TMAO molecule per NIPAm unit is necessary to reduce the number of hydrogen bonds between PNIPAm and water below a critical threshold that leads to (1) a stabilisation of the collapsed state which consequently implies (2) the suppression of gelation.

Our results validate recent work on the role of TMAO on the phase behavior of PNIPAm nanogels^38^, ^41^ and indicate the proposed stabilisation of the collapsed state due to preferred interaction between water and TMAO. Nevertheless, a molecular level understanding of the interplay between TMAO‐NIPAm interactions, in particular for different nanoparticle volume fractions, is still lacking which may motivate further theory and simulations studies on dense nanogel states.

## Experimental Section

### Samples

We studied colloidal core‐shell nanoparticles consisting of silica spheres coated with PNIPAm. The PNIPAm shell had been cross‐linked using 4 wt.% methylene bisacrylamide. Details of the synthesis are given in Refs. [20, 57]. The stock solution with approximately 16.5 wt.% silica‐PNIPAm particles was diluted 1 : 1 in volume with TMAO solutions, resulting in TMAO concentrations *c* in the solvent of 0.11 mol/L, 0.16 mol/L, 0.26 mol/L, 0.52 mol/L. The silica cores had a radius of 50 nm and a dispersity of 9 %. The size of the PNIPAm shell was measured by DLS (sample SP_1_ in [20]) and found to have a thickness of 50 nm at 20 °C and 25 nm in the collapsed state at 45 °C. This corresponds to an effective volume fraction of $\Phi_{\mathrm{eff},20}=0.141$ in the swollen state at 20 °C and $\Phi_{\mathrm{eff},45}=0.059$ at 45 °C. For calculation of structure factors, particle form factors were measured from a dilute dispersion $\left(\Phi_{\mathrm{FF}}\leq 0.01\right)$ . Before the experiment, the samples were filled into glass capillaries (Hilgenberg GmbH) with 2 mm diameter that have been vacuum‐sealed afterwards.

### Coherent small‐angle X‐ray scattering experiments

The X‐ray scattering experiment has been performed at beamline 8‐ID‐I at the Advanced Photon Source, Argonne National Laboratory (USA). We used a small‐angle X‐ray scattering (SAXS) geometry with a photon energy of 11 keV and a sample‐detector distance of 4.0 m. The capillaries were mounted in a SAXS chamber with adjustable temperature. The X‐ray beam was collimated to a size of 20 μm×20 μm. The scattered intensity was measured with a two‐dimensional LAMBDA 750 K (Large Area Medipix Based Detector Array) detector^58^, ^59^ at a frame rate of 2 kHz. The colloidal suspensions were measured between 20 °C and 45 °C with steps of 1 °C between 32 and 40 °C. After the desired temperature had been reached, an equlibration time of 600 s was added before the speckle patterns were measured. The total dose per spot was limited to $1.6\times 10^{4}$ Gy by accumulating series of 2000 speckle patterns which is well below the radiation damage threshold for the sample.^60^ Measurements were repeated at up to 20 spots on the sample in order to increase the statistical accuracy. Afterwards, the *g*_2_ functions have been averaged for each temperature.

## Conflict of interest

The authors declare no conflict of interest.
